# Editorial: Insights in pediatric urology

**DOI:** 10.3389/fped.2024.1517709

**Published:** 2024-11-27

**Authors:** Miguel Castellan, Lisandro Piaggio

**Affiliations:** ^1^ Department of Pediatric Urology, Nicklaus Children’s Hospital, University of Miami, Miami, FL, United States; ^2^ Departamento de Ciencias de la Salud, Universidad Nacional del Sur, Bahía Blanca, Argentina

**Keywords:** robotic pediatric urology, laparoscopic pyeloplasty (LP), splenogonadal fusion (SGF), urodynamic pediatric, heminephrectomy

**Editorial on the Research Topic**
Insights in pediatric urology

This collection of articles presents a diverse range of contributions to Pediatric Urology. These articles delve into the management of various pediatric urological conditions, offering valuable insights and contributing to the advancement of knowledge in this area.

Minimally invasive surgeries, such as laparoscopic or robotic pyeloplasty, are commonly used to treat ureteropelvic junction obstruction (UPJO) in children (1). These procedures have reported success rates exceeding 94%. Li et al. compared the effectiveness of robot-assisted single-port-plus-one pyeloplasty (RSPY) and laparoscopic single-port pyeloplasty (LSPY) in treating children with UPJO. Surgical incisions were similar except for the absence, in the LSPY group of patients, of an extra 8-mm incision in the flank to place a trocar for the 4th robotic arm. RSPY was associated with shorter intraoperative anastomosis and overall operation times compared to LSPY (*P* < 0.05). Both groups achieved satisfactory outcomes with no significant difference in postoperative complications. While RSPY offers advantages in surgical time, intraoperative anastomosis time, and shorter hospitalization, its higher cost may limit its widespread adoption. They recommend careful patient selection to optimize the benefits of RSPY.

Laparoscopic pyeloplasty (LP) is a minimally invasive surgical technique to correct ureteropelvic junction obstruction (UPJO). While it's a common procedure for older children, its feasibility in very young infants, especially those under six months old, has been questioned (2). A study by Langreen et al. addressed this concern by investigating the outcomes of LP in 49 infants younger than six months. They compared these results to 42 infants who underwent traditional open pyeloplasty (OP). Notably, approximately 20% of the LP group were less than six weeks old at the time of surgery, making this one of the youngest series of patients to undergo minimally invasive pyeloplasty. The mean age of this group was 2.6 months, with the youngest patient being just one week old. The study found that the revision rate for pyeloplasty was 8% in the LP group and 14% in the OP group for infants aged 0–6 months. This suggests that LP can be a viable option for treating UPJO in even the youngest of patients, potentially offering the advantages of minimally invasive surgery, such as reduced pain, shorter hospital stays, and faster recovery times.

In pediatric patients with duplex kidneys undergoing upper pole heminephrectomy is one of the options when treating an obstructed upper pole (3). Wang et al., reported on the risk factors for postoperative adverse events in 53 children with duplex kidneys who underwent an upper pole heminephrectomy. In this retrospective study, they used a univariate and multivariate analysis, and the incidence of postoperative adverse events was 30.2% and the reoperation rate was 9.4%. The presence of upper renal ureterocele, as well as the presence of accessory renal artery type as the upper kidney's blood supply artery increased the risk of postoperative adverse events. They were an independent risk factors for postoperative adverse events.

Urodynamic studies (UDS) are commonly used to evaluate lower urinary tract function in pediatric patients with neurogenic bladder or other related conditions (4). Yang et al. conducted a single-center study in Chengdu, China, to evaluate a Y-shaped connection device for pediatric patients undergoing UDS with indwelling catheters. The study involved 45 children with a mean age of 13 years, comparing the Y-shaped device to a standard UDS procedure. Compared to traditional traces, the trace curve obtained with the Y-shaped connection device is thicker and exhibits more pronounced fluctuations. Regarding complications, 8 (17.8%) patients experienced bleeding during re-catheterization in the traditional UDS approach. In contrast, no patients experienced bleeding with the Y-shaped device. The results demonstrated that the Y-shaped device effectively measures the storage period while causing less pain and bleeding. This makes it a promising alternative for children requiring UDS, especially those with neurogenic bladder focusing on filling phase parameters.

The Shehata technique offers a vessel-sparing approach to treating high intra-abdominal testes. It involves a two-stage procedure, fixing the testis to the contralateral anterior superior iliac spine during the first stage and then mobilizing it into the scrotum during the second (5). Lin et al. reported successful outcomes in 20 patients with an average follow-up of 26.05 months. The study, conducted at Baoan Women's and Children's Hospital, found no testicular atrophy or other complications. They concluded that the Shehata technique is a viable option for treating high undescended intraabdominal testes that cannot be brought into the scrotum in a single procedure.

Splenogonadal fusion (SGF) is an uncommon congenital anomaly that can present as scrotal enlargement, often mimicking a testicular tumor (6). This condition should be considered in the differential diagnosis of scrotal masses, particularly when history suggests a long-standing, stable lesion. Luo et al. reported three cases of SGF and reviewed the relevant literature. All reported cases involved the left side. This study underscores the importance of recognizing SGF and highlights the role of preoperative imaging, such as ultrasound, in making an accurate diagnosis. SGF can also be presented as cryptorchidism. Laparoscopic surgery, either staged or single-stage, is a suitable approach for definitive management. By increasing awareness of SGF, clinicians can improve preoperative diagnosis and optimize treatment planning for patients with scrotal enlargement.

This research topic and its associated manuscripts collectively highlight contemporary approaches to various pediatric urological conditions. These contributions enhance our understanding and management of these conditions. We extend our gratitude to all contributors and hope that readers find these articles informative and beneficial.
